# Estimating HIV prevalence from surveys with low individual consent rates: annealing individual and pooled samples

**DOI:** 10.1186/1742-7622-10-2

**Published:** 2013-02-27

**Authors:** Lauren Hund, Marcello Pagano

**Affiliations:** 1 Department of Family and Community Medicine, University of New Mexico, 2400 Tucker NE, Albuquerque, NM 87106, USA; 2 Department of Biostatistics, Harvard School of Public Health, 677 Huntington Street, Boston, MA 02115, USA

**Keywords:** Group testing, HIV/AIDS, Pooled testing, Survey non-response

## Abstract

Many HIV prevalence surveys are plagued by the problem that a sizeable number of surveyed individuals do not consent to contribute blood samples for testing. One can ignore this problem, as is often done, but the resultant bias can be of sufficient magnitude to invalidate the results of the survey, especially if the number of non-responders is high and the reason for refusing to participate is related to the individual’s HIV status. One reason for refusing to participate may be for reasons of privacy. For those individuals, we suggest offering the option of being tested in a pool. This form of testing is less certain than individual testing, but, if it convinces more people to submit to testing, it should reduce the potential for bias and give a cleaner answer to the question of prevalence. This paper explores the logistics of implementing a combined individual and pooled testing approach and evaluates the analytical advantages to such a combined testing strategy. We quantify improvements in a prevalence estimator based on this combined testing strategy, relative to an individual testing only approach and a pooled testing only approach. Minimizing non-response is key for reducing bias, and, if pooled testing assuages privacy concerns, offering a pooled testing strategy has the potential to substantially improve HIV prevalence estimates.

## Introduction

Hiv prevalence estimates derived from national population-based surveys are often considered the gold standard of hiv prevalence estimation when non-response rates are low [1-4]. However, finding and obtaining a blood sample from all individuals surveyed is a considerable, if not almost impossible, challenge. Frequently, migrant or homeless populations are ignored and a large proportion of the sample does not consent to being tested, potentially inducing (unmeasured) bias in the hiv prevalence estimators [4].

In this paper, we discuss a method for promoting increased testing consent rates. Individual reluctance to test may be influenced by several factors, including those related to social stigma associated with hiv and difficulty in accessing treatment for some testing individuals [5,6]. While no consensus has been reached on reasons for test refusal or failing to return for test results, fear is a common theme in such studies [7], and there is evidence that those who are aware of their positive hiv status are less likely to consent to testing [8].

Additionally, the hiv testing protocol is an important factor in gaining test consent [9]. The method of asking for consent, specifically convincing survey participants of the importance of their contribution to fighting the hiv epidemic while assuaging concerns about privacy of test results, could be key in improving test consent rates. Previous studies have assessed the impact of anonymity in testing by examining testing rate trends following transitions from anonymous to name-based reporting; there is some evidence in the literature that eliminating truly anonymous testing would impact individuals’ decisions to test hiv, though the results are not consistent (see [10,11] and references within).

One option for estimating prevalence while preserving the nonidentifiability of individuals, at the cost of greater uncertainty, is pooled testing [12], where individual samples are combined to form pooled samples. In this paper, we propose a testing protocol that supplements the presumably more informative individual testing with pooled testing. Each sampled individual is asked to provide a blood sample for disease testing, where the investigators (and, by choice, the individual as well) learn the disease status of the individual. If the individual rejects this testing option, we ask if he will provide a non-identifiable blood sample which will be combined with other samples in a pooled test and, in which case, *no one* knows this individual’s test result. If the individual does not consent to pooled or individual testing, then he is not tested for the disease, of course.

Ideally, by providing the pooled testing option, the amount of missingness in the sample is substantially reduced. Pooled testing strategies are frequently used in practice [13-18], but to our knowledge, have never been discussed in the context of improving survey response rates by varying the testing options. In this paper, we propose such an estimator and study its analytical properties. First, we discuss testing consent rates in hiv prevalence estimation surveys and give examples of when non-response bias is an issue in such surveys. We then discuss how to implement a new pooled testing strategy and propose an estimator for prevalence based on this testing strategy, assuming perfect sensitivity and specificity of the test. We present results from a simulation study examining small sample properties of this estimator and illustrating the importance of pool size choice in such a survey design.

## Missingness in HIV prevalence estimation surveys

Surveys designed to estimate hiv prevalence can have low testing consent rates, and test refusal is potentially associated with risk of hiv infection. Depending on what is driving test refusal in the population, missingness in a sample may induce bias in the estimator of prevalence [4]. Reviews of national hiv prevalence surveys have concluded that, while those who refuse testing may have a higher hiv prevalence, bias induced by missingness is usually negligible because response rates are on average sufficiently high [2,3]. However, the authors make strong assumptions about missingness patterns in the survey and also reference many surveys in which response rates are low enough that it is difficult to believe that bias in prevalence estimators is negligible. For instance, the hiv testing consent rate is 62.2% in men and 68.2% in women in the most recent national South African survey [19], and consent rates are even lower in the longitudinal hiv surveillance survey in rural KwaZulu Natal, South Africa, described in [20].

A taxonomy of the types of patterns of missingness is useful for analysis [21]. When missingness is at random, survey calibration techniques (such as weight-class adjustments, poststratification, and imputation) allow for adjustment of prevalence estimators to remove bias [22]. All such methods depend on the assumption of missing at random, which states that conditional on covariates, the outcome of interest (hiv status) is independent of the missingness mechanism (test refusal). Many studies have shown that hiv test results are not missing completely at random (see [7] and references within); further, assuming missingness is at random is a strong and untestable assumption.

When asking individuals to consent to hiv testing, regardless of how much covariate information is available on these individuals, one could reasonably infer that missingness is nonignorable, is associated with disease status, and cannot be completely explained by individual characteristics. For instance, individual covariate information is likely to be unreliable or sparse when dealing with sensitive topics, such as risky sexual behavior, fidelity, or drug use [23]. Sensitive issues such as partaking in risky sexual behavior are of course associated with hiv status, and studies suggest that there are inconsistencies in reporting of sexual behavior in Demographic Health Surveys (dhs) [24,25]. Further, using dhs data from Zambia, one recent study concluded that models based on observed covariates (i.e. assume missingness is at random) are insufficient to correct for selection bias in hiv prevalence estimation surveys, though this study relied on strong, untestable modeling assumptions [26]. Such studies reiterate that the best way to ensure unbiased prevalence estimates is through eliminating non-response.

When missingness is *not* at random, the (heuristically) most conservative range of estimates for hiv prevalence in a sample calculates the lower bound for prevalence by assuming that all non-responders are hiv negative and the upper bound by assuming all non-responders are hiv positive. Such plausibility bounds are obviously very wide when the proportion of non-responders is high but are also arguably the most honest bounds for our certainty regarding the sample prevalence estimates. Specifically, if only a fraction *r* of the sample responds to the survey, the prevalence of hiv in the sample is *p*=*rp*_*R*_+(1−*r*)*p*_*N*_, where *p*_*R*_ is the sample prevalence in the responders and *p*_*N*_ denote sample prevalence in the non-responders. Since we only know that *p*_*N*_ is between 0 and 1, the lower bound for prevalence in the sample is *rp*_*R*_ and the upper bound is *rp*_*R*_+(1−*q*). The width of this interval is 1−*r*, illustrating the importance of maximizing *r* in the presence of nonignorable missingness.

As an example, consider the 2004 dhs survey in Malawi [27]. The overall response rate for hiv testing was 70% in women and 63% in men. Of those interviewed by health workers, 22% refused hiv testing; the remainder of the non-response was driven by inability to locate sampled individuals for testing. In the Lilongwe district, the response rate was only 39%, with 49% of subjects refusing hiv testing and the rest unable to be located. The observed prevalence of hiv for the Lilongwe district was 3.7% with 95% CI [sic] (1.0%, 6.4%), whereas the observed prevalence in the rest of the country was 13.2% with 95% CI [sic] (12.3%, 14.2%). The hiv prevalence estimates for Lilongwe were deemed “implausibly low” and prevalence was imputed for everyone in the district of Lilongwe based on demographic information obtained in the household survey. The imputed prevalence for the Lilongwe district was estimated at 10.3% with 95% CI [sic] (9.3%, 11.3%).

Consider the conservative plausibility bounds mentioned above for the Lilongwe district. There were 500 individuals eligible for hiv testing in the district of Lilongwe, but only 193 of those eligible consented to hiv testing. Based on this information, we deduce that about seven out of the 193 consenters were hiv positive. If we assume all 307 non-consenters were hiv negative, a lower bound for hiv prevalence is 1.4% with 95% CI (0.4%, 2.4%); likewise, if we assume all 307 non-consenters were hiv positive, an upper bound for hiv prevalence is 62.8% with 95% CI (58.6%, 67.0%). By taking the lower confidence bound when we assume all non-responders are hiv negative and the upper confidence bound when we assume all non-responders are hiv positive, we can obtain the most conservative plausibility bounds at the 95% confidence level. In the Lilongwe case, the heuristic “plausibility bounds” for the prevalence of hiv are (0.4%, 67.0%), which now includes the national prevalence estimate for hiv in Malawi. While no one would ever present such wide plausibility bounds, these extreme bounds show the true amount of certainty we have when we know nothing about non-responders. The Lilongwe example illustrates the dangers of high non-response in an hiv prevalence estimation survey.

In many hiv prevalence surveys, non-response rates may be modest, and missing at random corrections will suffice for producing nearly unbiased HIV prevalence estimates. For instance, [3] list nonresponse rates by country and sex for DHS/AIS surveys; response rates exceeded 90% for both males and females in the Rwanda and Cambodia 2005 AIS surveys. Many other countries also retained high testing rates. However, in locations such as Malawi and South Africa, where prevalence and non-response are both high, alternative testing strategies are a viable tool for decreasing non-response and improving prevalence estimates.

## Testing logistics and pool size

In standard hiv testing surveys, individuals are only asked to consent to an hiv test once. Using a pooled testing option, we offer two opportunities to consent to hiv testing. For those who select the non-identifiable pooled testing option, individual blood samples are pooled with *k*−1 other blood samples (*k*>1), and only the test result of the pool is known to anyone. We delay discussion about appropriate choice of *k* to below. Though we anticipate that some will still refuse both individual and pooled hiv testing, the intent is to lower missingness in the sample (and the associated inherent bias in the estimator) by including individuals who refuse individual testing but are willing to provide a sample for pooled testing. We propose a combined individual and pooled testing prevalence estimator, for which privacy is preserved but prevalence can be estimated more accurately than when using only those willing to submit to individual testing.

Many possibilities exist for adapting testing protocols to include a pooled testing option. For instance, participants could first be asked to take a rapid test and learn their status; alternatively, standard ELISA blood tests could be administered, with the option of obtaining results at a later date. For those who were not interested in either method of individual testing, the pooled testing option would be explained. A simple illustration of how pooling works might aid in understanding how the protocol works (for instance, pouring together vials of different colored water into a cup).

Preserving privacy of the pooled testers is a primary concern in our protocol. If a pool tests negative, we know the test results of individuals in the pool (negative) within the bounds of the sensitivity of the testing kit. Presumably, individuals are not as concerned with the confidentiality and identifiability of negative test results, and we are not concerned with this situation. If a pool tests positive, individual test results in the positive pool are non-identifiable for pools of size 2 or bigger. Of course, the issue of trust is important; those carrying out the survey need to convince those surveyed that their privacy requests be respected if we wish to lower the refusal rate as much as possible. Furthermore, ethical non-identifiability for positive pools may mandate larger pool sizes.

If a pool tests positive, the probability that an individual is positive is *p*/(1−(1−*p*)^*k*^) in a population with prevalence *p*. For instance, when the population prevalence is 20%, the probability that an individual in a positive pool is hiv positive is 1 when for pool size *k*=1 (individual testing), 0.56 when *k*=2, 0.41 when *k*=3, 0.34 when *k*=4, 0.30 when *k*=5, 0.27 when *k*=6, and 0.25 when *k*=7. Since the population prevalence is 20%, without testing at all, the probability a person is infected is 20%. As *k* increases, the probability that an individual tests positive given the pool tests positive approaches the population prevalence. Thus, as pool size and prevalence increase, we gain less additional information about the disease status of individuals in a pool when the pool tests positive.

However, using pool sizes that are too large decreases accuracy of the pooled testing estimator (we further discuss the implications of pool size below). The key idea in this confidentiality protection problem is “to balance the need for confidentiality protection with legitimate needs of data users” [28]. The United States’ Federal Commission for Statistical Methodology lays out threshold rules for identifiability of survey responses for tabular data within U.S. Agencies; generally, at least 3-5 responses per cell are required for non-identifiability, but this minimum choice of responses per cell often varies with the sensitivity of the information and potential for disclosure [29]. In order to use the pooled samples, pool size must be carefully selected by balancing the precision of the pooled estimator with the ethical restraints imposed by nondisclosure of individual test information.

In this paper, we consider pool sizes to be between 3 and 7. While a smaller pool size will always result in a better estimator, pool size must be sufficiently large to protect the confidentiality of the testers; we assume ethical limitations would never mandate having a pool size larger than 7 and use this as our maximum pool size. In settings with low prevalences and cost constraints, higher values of *k* would be warranted.

## Framework for combining individual and pooled test results

To construct an estimator for hiv prevalence based on the pooled testing strategy, we assume *n* individuals are randomly sampled from a large population with hiv prevalence *p*. Further, we assume that the hiv test is a perfect test, i.e. the sensitivity and specificity are 1 (we comment further on this assumption in the Conclusion).

The sample can be partitioned into three separate groups: 1) those who consent to testing for a disease, 2) those who only consent to unidentifiable pooled testing, and 3) those who refuse testing altogether. The prevalence in each of these three groups may differ. To estimate prevalence, we can collapse across these partitions. For a population with prevalence *p*,

$$p=r_{1}p_{1}+r_{2}p_{2}+r_{3}p_{3},$$

where *r*_*i*_ is the proportion of the population in testing consent group *i* and *p*_*i*_ is the prevalence of hiv in group *i*. Individuals with *i*=1 consent to individual testing, with *i*=2 consent to pooled testing only, and with *i*=3 do not consent to test.

Note that we can never know *p*_3_, the prevalence in the non-consenters, and any estimator of *p* will always be biased unless everyone consents (*r*_3_=0); or we adjust the prevalence estimator based on some known and identifiable structure on *p*_3_, such as *p*_3_=*p*_2_. However, we can estimate the probability of having hiv given that one consents to test, denoted *p*_*T*_. Conditioning on the subset of the population who consents to some form of testing, we define *q*_1_ as the proportion of the population who consents to individual testing; and *q*_2_ as the proportion who consents to pooled testing. We estimate hiv prevalence in the testing consent group as *p*_*T*_=*p*_1_*q*_1_+*p*_2_*q*_2_.

We estimate *q*_1_,*q*_2_, and *p*_1_ using sample quantities from the data (e.g. *q*_1_ is the fraction of individuals who test individually, and *p*_1_ is the fraction of the individual testers who are hiv positive). Because of the desire to preserve anonymity, we cannot directly calculate the fraction of HIV positive individuals in the pooled testing population, *p*_2_. Rather, we observe the number of pools that test positive, denoted *Z*.

Among the pooled testers, we model *Z* using a binomial distribution, with sample size *n*_*p*_ (the number of pools) and proportion of positive pools *p*_*z*_=1−(1−*p*_2_)^*k*^, where *k* is the number of samples per pool; intuitively, the expression for *p*_*z*_ is equivalent to 1-P(all samples in a pool test negative). Inverting the formula for *p*_*z*_, it follows that *p*_2_=1−(1−*p*_*z*_)^1/*k*^. Define ${\hat{p}}_{z}=Z/n_{p}$. We can estimate the prevalence in the pooled testing population as ${\hat{p}}_{2}=1-{\left(1-{\hat{p}}_{z}\right)}^{1/k}$. This estimator is unbiased in large samples [13], and, for a fixed sample size, the variance of ${\hat{p}}_{2}$ increases as the pool size *k* increases.

We estimate *p*_*T*_ using the sample quantities from the data, ${\hat{p}}_{T}={\hat{q}}_{1}{\hat{p}}_{1}+\hat{q_{2}}{\hat{p}}_{2}$; we refer to ${\hat{p}}_{T}$ as the combined prevalence estimator. Further, ${\hat{p}}_{T}$ is asymptotically normally distributed with mean *p*_*T*_ and variance:

$$\begin{array}{l} \;m*\text{var}({\hat{p}}_{T})=q_{1}p_{1}(1-p_{1})+q_{2}\frac{1}{k}{\left(1-p_{2}\right)}^{2}\left({\left(1-p_{2}\right)}^{-k}-1\right)\\ \;+q_{1}q_{2}{\left(p_{1}-p_{2}\right)}^{2}, \end{array}$$

where *m* is the total number of testing individuals in the sample. For more details, see Appendices 3-6 in Additional file 1. We can obtain a variance estimate for ${\hat{p}}_{T}$, $\hat{\text{var}}({\hat{p}}_{T})$, by plugging in the sample estimates into the above equation. Therefore, we can define a 100(1−*α*)*%* Wald-type confidence interval for ${\hat{p}}_{T}$ as ${\hat{p}}_{T}\pm z_{1-\alpha /2}\sqrt{\hat{\text{var}}({\hat{p}}_{T})}$.

## Comparing testing strategies with large sample sizes

To assess the properties of our combined testing strategy (assuming a perfect test), we consider relative low, moderate, and high population prevalence settings where individual testing consent rates are low. In the low prevalence setting, we assume the prevalence in the individual testers is 5% and the prevalence in the pooled testers is 10%; in the moderate setting, prevalence in individual testers is 15% and in pooled testers is 20%; and in the high prevalence setting, prevalence in the individual testers is 20% and in the pooled testers is 30%. These settings are important to keep in mind and are referenced throughout the paper as the low, moderate, and high prevalence settings. We assume that the sub-population that consents to individual testing constitutes 60% of the total testing population and the sub-population that will only contribute a sample for pooled testing constitutes 40% of the population.

We contrast the combined pooling and individual testing estimator with alternatives using asymptotic mean-squared error (mse), defined as the sum of the squared-bias and the variance of the estimator when sample sizes are large. It is important to balance both precision and accuracy when contrasting estimators, and we select mse because it incorporates bias and variance. Later, we address the scenario when sample sizes are not large and finite-sample bias can arise.

First, we contrast the combined estimator to the prevalence estimator resulting from only offering individual testing. If the pooled testing option is omitted and individual testing is the sole testing option, an estimate of the prevalence in the population is ${\hat{p}}_{1}$, the estimated hiv prevalence in the individual testing population. Assuming for now that *r*_3_=0, the bias in ${\hat{p}}_{1}$ is *p*_1_−*p*=*r*_2_(*p*_1_−*p*_2_), which is non-zero when *p*_1_≠*p*_2_ and *r*_2_≠0. However, even if *p*_1_≈*p*_2_, the estimator using pooled samples will usually have a smaller variance than the estimator that does not incorporate pooled testing, as long as a sufficient proportion of the population consents to pooled testing.

**Figure 1:**
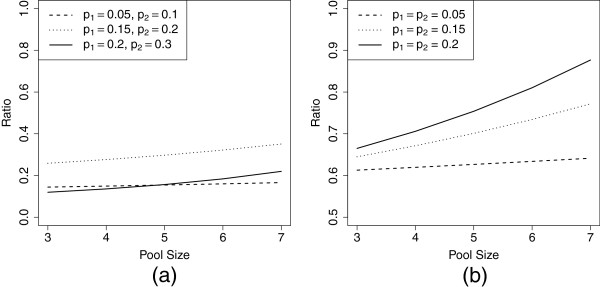
**Comparing the asymptotic properties of the combined estimator to the individuals-only estimator.** Ratio of the asymptotic mse for the combined estimator to the ratio of the asymptotic mse for the estimator using only individuals in the low, moderate, and high prevalence settings for two scenarios: (**a**) pooled testers have a higher prevalence than individual testers, *m*=1000; (**b**) the prevalence in the pooled testers equals that in the individual testers (this ratio is independent of *m*). The combined estimator always has lower mse than the individuals only estimator in these settings.

Since the combined estimator is asymptotically unbiased, the asymptotic mean-squared error of the estimator is identical to the variance of the estimator. The estimator using only individual testers has mse equal to the sum of the variance of $\hat{p_{1}}$ and the square of the bias of the prevalence estimator when the pooled testers are excluded. The ratio of the mse using the pooled strategy versus the mse using individuals only is always less than one when the pool size is less than 7 for the low, moderate, and high prevalence settings (Figure 1), indicating that the combined estimator outperforms the estimator using only individuals. Indeed, in the situations in which pooled testers have a higher prevalence than individual testers, the mse ratio ranges between 0.1 and 0.4, and the combined estimator provides substantial improvement over the estimator ignoring pooled testers. Even when the prevalence is the same in the pooled and individual testing populations, the mse ratio ranges between 0.6 and 0.85, and the combined estimator still outperforms the individuals-only estimator.

On the other hand, only offering pooled testing to everyone in the sample, as suggested in [12], is cheaper than offering an individual and pooled testing option, because fewer tests are performed. For instance, we could design a study which *only* offers a pooled testing option and estimate prevalence using the maximum likelihood estimator for pooled samples discussed previously. The prevalence estimator resulting from pooling everyone is asympotitcally unbiased, because we include the entire testing population.

**Figure 2:**
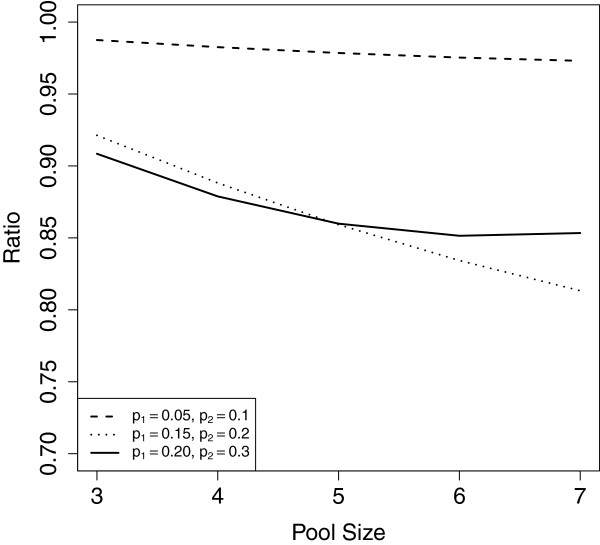
**Comparing the asymptotic properties of the combined estimator to the pooling-only estimator.** Ratio of the mse for the combined estimator to the ratio of the mse when everyone is offered pooled testing, as a function of pool size for the low, moderate, and high prevalence settings when pooled testers have a higher prevalence than individual testers. The combined estimator always has lower mse than the estimator where everyone is offered pooled testing in these settings.

Testing using the combined estimator results in a smaller asymptotic mse than the estimator which only offers pooled testing (Figure 2), assuming the sample size is the same for both estimators. The mse for the combined estimator is 10% less than the mse for the pooled testing only estimator in the moderate and high prevalence settings, with less reduction in mse in the low prevalence setting. The combined estimator provides an improvement in mse because the variance of the pooled prevalence estimator always decreases as the pool size decreases; intuitively, individual test results provide more information than pooled test results on the same number of people, so providing an individual testing option is optimal. Further, if everyone is offered pooled testing, individual results are no longer available to those who are interested in learning their hiv status and thus may be unethical [30]. And lastly, the survey protocol we suggest gives individuals two opportunities to consent to testing (pooled or individual), rather than only asking individuals to test once as in the pooled-testing only design, which could help increase consent rates. Therefore, having both pooled and individual testing options is advantageous.

### Assessing the finite sample properties of the combined estimator

Pooling has its limitations that are a function of prevalence. When the prevalence is high, then, to be informative, the pools must be so small as not to have all the pools test positive [13,31]. [18] investigate the properties of pooled estimators in high prevalence settings. On the other hand, to retain anonymity, the pool sizes cannot be too small. Statistically, pooled estimators are potentially unstable when the prevalence in the pooled-sample population (*p*_2_) is high or when the number of individuals consenting to pooled testing is small.

In the case of most diseases that are not extremely rare, such as hiv, the disease prevalence is typically high enough that some pools will test positive, and we are not concerned with zero pools testing positive. However, in moderate to high prevalence settings, the probability that all pools will test positive must also be addressed. This probability is *P*(*Z*=*n*_*p*_)=(1−(1−*p*_2_)^*k*^)^*n*^_*p*_, which decreases as *n*_*p*_ increases and/or *k* and *p*_2_ decrease. Therefore, choosing a sufficiently small pool size *k* and obtaining a sufficiently large number of pools *n*_*p*_ are necessary to ensure that the estimate of the population prevalence in the pooled testing group is reasonable. Note that the lower bound for *k* is determined by how large the pools should be to assuage concerns about identifiability of test results.

Pooled prevalence estimators are biased in finite samples [13], and consequently, ${\hat{p}}_{T}$ is only asymptotically unbiased (see Appendix 5 in Additional file 1). While replacing an estimator with a jackknifed version of the estimator typically reduces finite sample bias [32-34], in simulation, we find that the jackknife estimator provides little improvement over the original estimator (results not shown). Other suggestions for bias correction to the pooled prevalence estimator have been suggested [35]. For instance, in high prevalence settings, [18] propose a double grouping estimator.

Burrows [31] suggests the simple estimator (subsequently referred to as the Burrows estimator):

$${\tilde{p}}_{2}=1-{\left[\frac{2\text{kZ}+k-1}{2kn_{p}+k-1}\right]}^{1/k}.$$

**Figure 3:**
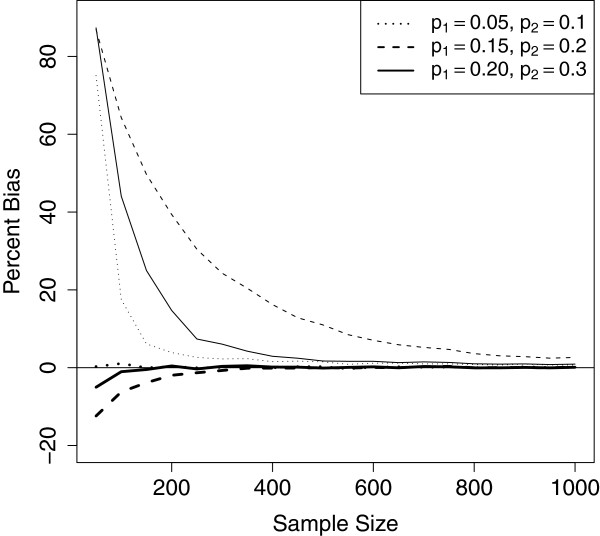
**Percent bias in the combined estimator.** Percent bias in the MLE estimator ${\hat{p}}_{T}$ (thin lines) and the Burrows estimator ${\tilde{p}}_{T}$ (bold lines) for pool size *k*=7 as a function of sample size for low, moderate, and high prevalence settings. Using the Burrows estimator results in a substantial reduction in finite sample bias.

We can use the Burrows estimator to define a new prevalence estimator ${\tilde{p}}_{T}$, which is constructed by substituting ${\tilde{p}}_{2}$ for ${\hat{p}}_{2}$ in the combined estimator. This new estimator ${\tilde{p}}_{T}$ has much smaller finite sample bias than ${\hat{p}}_{T}$ in small samples. In Figure 3, we plot the percent bias in the prevalence estimator for ${\hat{p}}_{T}$ and ${\tilde{p}}_{T}$ for pool size *k*=7 (the size for which we see the greatest finite-sample bias). The original estimator ${\hat{p}}_{T}$ always overestimates the prevalence, with the severity of the bias decreasing as the sample size increases. The Burrows estimator ${\tilde{p}}_{T}$ has negligible bias, even for sample sizes as small as 100. Consequently, we recommend using ${\tilde{p}}_{T}$ in practice rather than ${\hat{p}}_{T}$.

**Figure 4:**
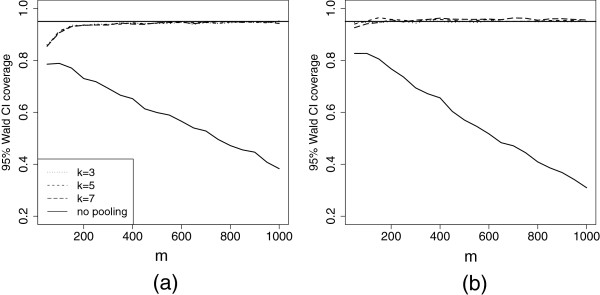
**Confidence interval coverage for the combined estimator.** 95% confidence interval coverage for ${\tilde{p}}_{T}$ as a function of sample size calculated using various pool sizes in the (**a**) low and (**b**) high prevalence setting as a function of the sample size.

In a simulation study, we evaluate maximum pool sizes and minimum number of pools such that the bias and standard error of ${\tilde{p}}_{T}$ are small and the 95% Wald confidence interval coverage of ${\tilde{p}}_{T}$ is near 0.95. Individuals who do not consent to testing at all are ignored throughout the simulations. Simulation parameters are chosen to reflect low, moderate, and high prevalence settings which have low testing consent rates for individuals, as described previously. We perform the simulation study for pool sizes 3, 5, and 7 (with 5,000 iterations each). Wald 95% confidence interval coverage is shown in Figure 4 for the low and high prevalence settings (the moderate setting was similar, but results are not shown). The 95% Wald confidence interval performs well for the combined estimator, with coverage lingering around 95% for moderate sample sizes. The confidence interval coverage drops below 60% very quickly when the pooled testers are ignored. As in the Lilongwe example, confidence intervals are misleading when selection bias exists in the sample.

**Figure 5:**
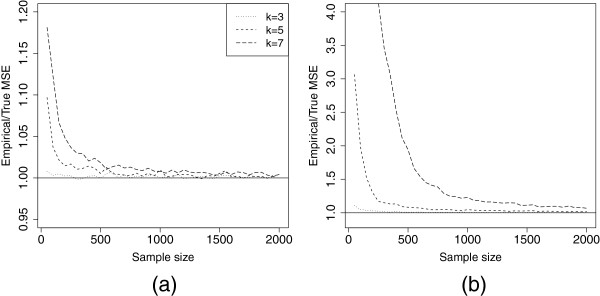
**Assessing the MSE of the combined estimator in simulation.** Plot of the ratio of the empirical to the true mse of the combined estimator as a function of sample size for the (**a**) low and (**b**) high prevalence settings. When asymptotic results are valid, this ratio will be close to one.

In small sample sizes for the moderate and high prevalence settings, the empirical standard error for the combined estimator is much larger than the derived standard error (results not shown), due to the fact that all of the pools test positive in a substantial proportion of the simulation runs. The derived large-sample standard error is not valid when all pools test positive, and, in such settings, using the pooled prevalence estimator in practice is not advised. Further, finite sample bias is problematic in small sample sizes when prevalence is moderate to high. Before using the asymptotic normality and variance formula for the combined estimator, it is important to know how many pooled testers are required for these asymptotics to be valid. In order to assess when the large-sample asymptotics hold and the combined prevalence estimator is valid, we calculate the ratio of the empirical mse and the asymptotic mse (see Figure 5 for the low and high prevalence settings). The asymptotic mse is described above, and the empirical mse is defined as the square of the average empirical bias in the combined estimator added to the empirical variance of the combined estimator in the 5000 simulations. Since both empirical variance and bias should be higher than the asymptotic variance and bias in finite samples, this ratio should provide a good metric for gauging the validity of our estimator. When this ratio is less than 1.05, we declare the estimator to be valid.

Table 1 provides suggestions as to minimum sample size and pool size required in the low, moderate, and high prevalence settings in order to obtain a valid estimator. We recommend not using pool sizes over 5 (preferably 3) in the high prevalence setting.

**Table 1:** Recommended sample sizes

**Pool size**		**Prevalence setting**	
	**Low**	**Moderate**	**High**
3	50 (20)	50 (20)	150 (60)
5	100 (40)	200 (80)	700 (280)
7	200 (80)	500 (200)	>2000 (>800)

Sample size (number of pooled testers) required for an empirical mse/true mse ratio <1.05.

## Conclusion

When investigators designing a disease prevalence survey anticipate high refusal rates for individual testing due to disease stigma, offering a pooled testing option and combining pooled and individual sample results has the potential to improve prevalence estimates. Ideally, everyone sampled will consent to either individual or pooled testing. In practice, we anticipate that some individuals will refuse to participate. In Appendix 1 in Additional file 1, we propose several potential adjustments to the prevalence estimator to account for the refusers. We note that the prevalence in the group that refuses to test altogether cannot be estimated without making strong modeling assumptions.

The proposed testing strategy addresses non-response rates in high prevalence, high non-response scenarios. In lower prevalence settings, pooled testing becomes more efficient and higher values of *k* are acceptable. In settings with high response rates, the combined pooled and individual testing strategy is not recommended, as the additional logistics and cost of implementation would not outweigh the small increase in the accuracy of the prevalence estimates.

We have assumed that, for a given survey, the pool size *k* does not vary. In high prevalence scenarios where it is possible that all pools might test positive, allowing *k* to vary could substantially improve the pooled estimator. For instance, [17] partition individuals by risk when forming groups. The form of the estimator for the prevalence among pooled testers would change in this setting and would no longer have a clean form.

Acquiring blood samples for pooled testing also allows the investigator to compare the prevalence in the individual testing population (*p*_1_) with the prevalence in the pooled testing population (*p*_2_). A test of the hypothesis that *p*_1_=*p*_2_ is simple to construct. This hypothesis test and a corresponding 95% CI for (*p*_1_−*p*_2_) can help determine the extent of selection bias in the sample. Evidence that the consenting and part of the refusing populations are not different with respect to disease status is valuable for generalizability of results to the entire population. Note that this is an association test which does not take any covariates into account, though the test could be conducted within strata if sample sizes are large enough.

Techniques have also been developed for regression analyses of disease status on covariates when blood samples are pooled [36-39]. Future work should investigate extending this testing strategy to facilitate regression modeling with the individual and pooled test results. Though we do not want to identify individuals within pooled samples, constructing pools that are homogeneous with respect to the covariates of interest increases the precision of the regression coefficient estimates [36]. Non-random missingness in covariates would likely pose an additional complication in designing a testing strategy to facilitate regression modeling.

Our proposed estimator above assumes a perfect test, but extending the estimator to imperfect tests is straightforward, as shown in [13], insofar as sensitivity and specificity do not vary with pool size. Sensitivity and specificity are generally high for hiv tests. However, if sensitivity and specificity are not close to 1, the merits of this testing strategy should be re-evaluated; imperfect tests can compromise the applicability of pooled testing in high prevalence scenarios [18]. Details about how to extend the estimator for imperfect tests are included in Appendix 2 in Additional file 1. Additionally, if sensitivity and specificity are a function of the pool size, the pooled test is subject to the ’dilution effect,’ substantially complicating prevalence estimation [40]. Future work should investigate extending this testing strategy to account for the dilution effect.

Many testing protocols are currently being used in hiv surveillance programs which aim to optimize efficiency and retain anonymity. There exists an ongoing debate about the ethics of unlinked anonymous testing (uat) [30,41,42]. In sentinel populations such as pregnant women at anc clinics, uat without informed consent is a commonly used protocol. Blood samples that are obtained for routine tests are also tested for hiv without any informed consent and are not linked back to the individual in any way. As treatment becomes more available, the ethics of such testing procedures become more questionable, and our suggested protocol requires obtaining informed consent from the individual. Voluntary uat (or uat with informed consent) is a much more widely accepted testing protocol and is currently used in dhs surveys. Informed consent is obtained before testing blood for hiv, but test results are not linked back to the individuals and, those who test cannot learn their disease status. Our testing protocol bypasses any of the ethical issues associated with uat, as sampled individuals have three options: 1) test as an individual and learn their disease status, 2) test as an individual and do not learn their disease status, or 3) submit blood for pooled testing and do not learn their disease status.

Lastly, in selecting survey design parameters, namely pool size and total sample size, an *a priori* estimate of *r*_2_ is necessary. This proportion can be estimated by conducting a small pilot study in the population before the survey is conducted. In constructing our estimators, we assume the data were generated from a simple random sample. The methodology can be extended to stratified or cluster sampling surveys, insofar as pools are composed within the strata and a sufficient proportion of the sample consents to pooled testing within each stratum.

## Competing interests

Both authors declare that they have no competing interests.

## Authors’ contributions

LH performed statistical simulations and derivations. MP supervised simulation and derivations. Both authors wrote and reviewed the final manuscript.

## Supplementary Material

Additional file 1 Appendix for “Estimating HIV prevalence from surveys with low individual consent rates: annealing individual and pooled samples”. Click here for file
