# Supplementary material for: Estimating HIV prevalence from surveys with low individual consent rates: annealing individual and pooled samples
Source: Emerg Themes Epidemiol. 2013 Feb 27;10:2. doi: 10.1186/1742-7622-10-2 (PMC3649931; doi:10.1186/1742-7622-10-2)
Supplement: Additional file 1 — Appendix for “Estimating HIV prevalence from surveys with low individual consent rates: annealing individual and pooled samples”. [file 1742-7622-10-2-S1.pdf]

Appendix for "Estimating HIV prevalence from surveys with low individual consent rates: annealing individual and pooled samples"

Lauren Hund and Marcello Pagano

January 11, 2013

1 Adjusting for individuals who refuse testing

Ideally, in a disease prevalence estimation survey, all sampled individuals will consent to test, either as an individual or in a pool. However, in practice, we anticipate that a certain proportion of the population, q_3, will refuse testing altogether. Unless we can assume test status is missing completely at random, accounting for this missingness induced by test refusal is key to constructing an unbiased estimator of prevalence. As previously discussed, assuming data is missing at random may be a poor assumption in such settings. A more reasonable assumption might be that that those who refuse testing are more similar to those who consent to pooled testing than they are to those who consent to individual testing. With this motivation, we propose a weight-class adjustment (also called response propensity weighting) to the estimator to improve the precision of population prevalence estimates [1].

First, we discuss weight-class adjustments without using a pooled testing design. If we have obtained a simple random sample of the population, the simplest estimator for the prevalence is the number of disease positive individuals in a sample who consent to testing divided by the total number of consenters. This estimator relies on the assumption that those who consent to test are representative of the entire population,

In order to adjust the prevalence estimator for non-consent, we divide the sample of size n into j different strata, $j = 1, ..., J$. Denote the number of individuals sampled in the jth stratum as n_j, and assume that m_j individuals in stratum j consent to testing. We can weight each consenting individual in the sample by the inverse probability that they consent to testing. This method of propensity score weighting produces an unbiased estimator of prevalence when consenters and non-consenters within stratum j are alike with respect to HIV status (that is, there are no unmeasured confounders within stratum j). Using propensity score weighting, the adjusted prevalence estimate becomes: $\hat{p} = \sum_j (n_j/n)\hat{p}^j$.

Propensity weighting adjustments have been discussed frequently in the literature and have disadvantages

including inflating the variance when the weights are large [2, 3, 4]. Such a situation would occur when individuals in a given stratum are very unlikely to participate in a survey. Collapsing strata can be effective in reducing the impact of sparse data and large weights within a stratum if such a situation occurs. Note that rather than dividing the data into strata, propensity scores can be calculated using logistic regression and weights can be constructed based on predicted probabilities from a logistic regression, as employed in [5].

This propensity weighting framework extends naturally to the combined prevalence estimator, assuming that we can construct homogeneous pools based on the j strata. Construction of homogeneous pools is the primary challenge of implementing the weight-class adjustment correction. Choosing appropriate strata requires balancing the need for a sufficient number of pooled testers within each stratum to maintain confidentiality and obtain valid prevalence estimates as well as the need to incorporate a sufficient amount of information about the testers versus non-testers. Assuming we can construct such strata, we can use the weight class adjustment in two different ways: 1) weight everyone in the sample who consents to test by the inverse probability of testing within their respective stratum, or 2) weight only the pooled testers by the inverse probability of testing as a pooled tester, conditional on not testing as an individual. The first method of weight class adjustment assumes that non-testers are similar to testers (pooled or individual) within strata with respect to HIV status, whereas the second method assumes that non-testers are similar to pooled testers within strata. To choose the appropriate adjustment method, reasons for not consenting to test should be obtained from the sample when possible. For instance, if most people will not test because they dislike having blood drawn, then the first method might be more plausible. If hesitance of the pooled testers and non-testers is caused by suspicion of HIV positive status, the second method is more reasonable.

Simpler estimators could also be proposed without employing a weight-class adjustment, which may be more feasible in practice. For instance, one could assume the prevalence of HIV in the non-testers is equal to the prevalence within the pooled testing population and suggest $\hat{p} = \hat{p}_1\hat{q}_1 + \hat{p}_2(\hat{q}_2 + \hat{q}_3)$, which is potentially a better estimator than $\hat{p}_T$ for the prevalence in the population. Lastly, we could assume a linear trend exists between p_1, p_2, and p_3, and define a prevalence estimator as $\hat{p} = \hat{p}_1\hat{q}_1 + \hat{p}_2\hat{q}_2 + \hat{p}_3\hat{q}_3$ using linear extrapolation (*e.g.* $\hat{p}_i = a + bi$). These estimators need to be tested in practice before we can contrast their merits.

2 Extending the estimator for imperfect sensitivity and specificity

Let ϕ and ψ represent test sensitivity and specificity, respectively. The probability that an individual consenter tests positive is $p_1\phi + (1-p_1)(1-\psi)$. We assume that there is no dilution effect, and sensitivity and specificity are the same for pools as for individual tests. The probability that a pool tests positive is $(1-(1-p_2)^k)\phi + (1-p_2)^k(1-\psi)$. Note that we also make the relatively mild assumption that $\phi+\psi-1>0$. It follows that $\hat{p}_{1,\phi,\psi} = (X_1/Y_1 + 1 - \psi)/(\phi+\psi-1)$ and $Var(\hat{p}_{1,\phi,\psi}) = Var(\hat{p}_1)/(\phi+\psi-1)^2$. Define $\tilde{p}_z$ as Z/n_p when using the standard pooled prevalence estimator; and as $(Z+(k-1)/2k)/(n_p+(k-1)/2k)$ when the Burrows correction is used. In the pooled setting,

$$\hat{p}_{2,\phi,\psi} = 1 - \left(\frac{\phi - \tilde{p}_z}{\phi+\psi-1}\right)^{1/k}$$

when $1-\psi \le \tilde{p}_z \le \phi$; $\hat{p}_{2,\phi,\psi} = 0$ when $0 \le \tilde{p}_z \le 1-\psi$; and $\hat{p}_{2,\phi,\psi} = 1$ when $\phi \le \tilde{p}_z \le 1$. Also, asymptotic normality for $\hat{p}_{2,\phi,\psi}$ holds, where $Var(\hat{p}_{2,\phi,\psi}) = Var(\hat{p}_2)/(\phi+\psi-1)^2$. Therefore, when the sensitivity and specificity of a test are known, they are easily incorporated into the framework of the individual and pooled testing prevalence estimator, as $\hat{p}_{T,\phi,\psi} = q_1\hat{p}_{1,\phi,\psi} + q_2\hat{p}_{2,\phi,\psi}$ and $(\hat{p}_{T,\phi,\psi} - p_T)/(\hat{Var}_{\phi,\psi}(\hat{p}_{T,\phi,\psi}))^{1/2} \sim N(0,1)$, where $Var_{\phi,\psi}(\hat{p}_{T,\phi,\psi})$ is simple to calculate by using the same form of the variance as $\hat{p}_T$, but substituting $V_1/(\phi+\psi-1)^2, V_2/(\phi+\psi-1)^2$ for V_1, V_2 (see Appendix 5 in Additional file 1). Sample variance is calculated by substituting $\hat{p}_{1,\phi,\psi}, \hat{p}_{2,\phi,\psi}$ for $\hat{p}_1, \hat{p}_2$.

3 Notation for statistical derivations

Let $Y = (Y_1, Y_2, Y_3)$ be a random variable classifying individuals by their testing consent choices, $Y \sim Multinom(n, r_1, r_2, r_3)$, where $n = Y_1 + Y_2 + Y_3$. Specifically, Y_1 reflects the number who consent to individual testing; Y_2 reflects the number who do not consent to individual testing but consent to pooled testing; and Y_3 reflects the number who do not consent to test at all. Let X_1 number of HIV positive persons who consent to individual test, X_2 number of HIV positive persons who consent to pooled test, and X_3 = number of HIV positive persons who do not consent to test. We model $X_i|Y_i \sim Bin(Y_i, p_i), i = \{1,2,3\}$. We define $q_1 = r_1/(r_1+r_2)$ and $q_2 = r_2/(r_1+r_2)$. Restricting to the testing population only, $(Y_1, Y_2) \sim Multinom(m, q_1, q_2)$ or equivalently $Y_1 \sim Bin(m, q_1)$, where m is the total number of individuals

who consent to test in the sample (pooled or individual).

A natural estimator for p_T is $\hat{p}_T = \hat{p}_1\hat{q}_1 + \hat{p}_2\hat{q}_2$, where $\hat{q}_1 = Y_1/m$, $\hat{q}_1 = Y_2/m$, $\hat{p}_1 = X_1/Y_1$, and $\hat{p}_2 = 1-(1-\hat{p}_z)^{1/k}$. Conditional on Y_2, the asymptotic variance of $\sqrt{Y_2}\hat{p}_2$ is $(1-p_2)^2((1-p_2)^{-k}-1)/k$ [6]. Note that $\hat{q}_1, \hat{q}_2$, $\hat{p}_1$, and $\hat{p}_2$ are unbiased estimators of q_1, q_2, p_1, and p_2 respectively, as $m \to \infty$. We assume q_1, q_2, p_1, and p_2 are non-zero.

4 Asymptotic unbiasedness of $\hat{p}_T$

$$
\begin{aligned}
E(\hat{p_T}) &= E_Y(E(\tfrac{Y_1}{m}\tfrac{X_1}{Y_1} + \tfrac{Y_2}{m}\hat{p}_2|Y)) \\
&= E_Y(\tfrac{Y_1}{m}p_1 + \tfrac{Y_2}{m}p_2) \text{ as } m \to \infty \text{ because } \hat{p}_2 \text{ is unbiased asymptotically} \\
&= p_T \text{ as } m \to \infty
\end{aligned}
$$

5 Derivation of asymptotic variance of $\hat{p}_T$

Denote $V_1 = p_1(1-p_1)$ and $V_2 = \frac{1}{k}(1-p_2)^2((1-p_2)^{-k}-1)$.

$$
\begin{aligned}
Var(\hat{p_T}) &= \underbrace{E(Var(\hat{p_T}|Y))}_{a} + \underbrace{Var(E(\hat{p_T}|Y))}_{b} \\
a: E(Var(\hat{p_T}|Y)) &= E(Var(\tfrac{X_1}{m} + \tfrac{Y_2}{m}\hat{p}_2|Y)) \\
&= E(Var(\tfrac{Y_1}{m}\tfrac{X_1}{Y_1}|Y) + Cov(\tfrac{Y_1}{m}\tfrac{X_1}{Y_1}, \tfrac{Y_2}{m}\hat{p}_2|Y) + Var(\tfrac{Y_2}{m}\hat{p}_2|Y)) \\
&= E(\tfrac{Y_1^2}{m^2}Var(\hat{p}_1|Y)) + 0 + E(\tfrac{Y_2}{m^2}Var(\sqrt{Y_2}\hat{p}_2|Y)) \\
&= \tfrac{1}{m}(E(\tfrac{Y_1}{m}V_1) + E(\tfrac{Y_2}{m^2}V_2)) \text{ as } m \to \infty \\
&= \tfrac{1}{m}(q_1V_1 + q_2V_2) \text{ as } m \to \infty \\
b: Var(E(\hat{p_T}|Y)) &= Var(\tfrac{Y_1}{m}p_1 + \tfrac{Y_2}{m}p_2) \text{ as } m \to \infty \\
&= Var(\tfrac{Y_1p_1}{m}) + 2Cov(\tfrac{Y_1p_1}{m}, \tfrac{Y_2p_2}{m}) + Var(\tfrac{Y_2p_2}{m}) \text{ as } m \to \infty \\
&= \tfrac{1}{m}\left(p_1^2q_1(1-q_1) - 2p_1p_2q_1q_2 + p_2^2q_2(1-q_2)\right) \text{ as } m \to \infty \\
&= \tfrac{1}{m}(q_1q_2(p_1^2 - 2p_1p_2 + p_2^2)) \text{ as } m \to \infty \\
Var(\hat{p_T}) &= \tfrac{1}{m}(q_1V_1 + q_2V_2 + q_1q_2(p_1-p_2)^2) \text{ as } m \to \infty
\end{aligned}
$$

6 Sketch of asymptotic distribution of $\hat{p}_T$

Again denote $V_1 = p_1(1-p_1)$ and $V_2 = (1/k)(1-p_2)^2((1-p_2)^{-k}-1)$.

As $m \to \infty$, $\sqrt{mq_1}\hat{p}_1 \sim N(p_1, V_1)$ and $\sqrt{mq_2}\hat{p}_2 \sim N(p_2, V_2)$; $\sqrt{mq_1}\hat{p}_1$ and $\sqrt{mq_2}\hat{p}_2$ are independent as $m \to \infty$.

Additionally, as $m \to \infty$,
$\sqrt{m}(\hat{q}_1' - q_1, \hat{q}_2' - q_2)^T \sim N(0, \begin{pmatrix} q_1(1-q_1) & q_1(1-q_1) \\ q_1(1-q_1) & q_1(1-q_1) \end{pmatrix}$ [7].

Rewrite:

$$\sqrt{m}(\hat{p}_T - p_T) \quad = \quad \underbrace{\sqrt{m}(\hat{p}_1(\hat{q}_1' - q_1) + \hat{p}_2(\hat{q}_2' - q_2))}_{a} + \underbrace{\sqrt{q_1}\sqrt{mq_1}(\hat{p}_1 - p_1) + \sqrt{q_2}\sqrt{mq_2}(\hat{p}_2 - p_2))}_{b}$$

Note that, as $m \to \infty$, a:

$$\sqrt{m}(p_1(\hat{q}_1 - q_1) + p_2(\hat{q}_2 - q_2)) \sim N(0, q_1(1-q_1)(p_1^2 - 2p_1p_2 + p_2^2)).$$

We know that $\hat{p}_1 \xrightarrow{p} p_1$ and $\hat{p}_2 \xrightarrow{p} p_2$. Then, as $m \to \infty$,

$$\sqrt{m}(\hat{p}_1(\hat{q}_1 - q_1) + \hat{p}_2(\hat{q}_2 - q_2)) \sim N(0, q_1(1-q_1)(p_1^2 - 2p_1p_2 + p_2^2)).$$

and b:

$$\sqrt{q_1}\sqrt{mq_1}(\hat{p}_1 - p_1) + \sqrt{q_2}\sqrt{mq_2}(\hat{p}_2 - p_2) \sim N(0, q_1V_1 + q_2V_2).$$

Asymptotically, $\sqrt{q_1}\sqrt{mq_1}(\hat{p}_1 - p_1) + \sqrt{q_2}\sqrt{mq_2}(\hat{p}_2 - p_2)$ and $\sqrt{m}(\hat{p}_1(\hat{q}_1 - q_1) + \hat{p}_2(\hat{q}_2 - q_2))$ are independent. Therefore,

$$\sqrt{m}(\hat{p}_T - p_T) \sim N(0, V_{p_T}) \text{ as } m \to \infty$$

where $V_{p_T} = q_1V_1 + q_2V_2 + q_1q_2(p_1 - p_2)^2$.

7 Derivation of finite sample bias in p_T

$$
\begin{aligned}
E(\hat{p_T}) &= E_Y(E(\tfrac{X_1}{m} + \tfrac{Y_2}{m}\hat{p}_2|Y)) \\
&= p_T + E\left(\tfrac{Y_2}{m}\tfrac{k-1}{2(1-p_2)}var(\hat{p_2})\right) + O\left((\tfrac{m}{k})^{-\frac{3}{2}}\right) \\
&\approx p_T + \tfrac{k-1}{2mk}(1-p_2)^{1-k}(1-(1-p_2)^k) + O((\tfrac{m}{k})^{-\frac{3}{2}})
\end{aligned}
$$

References

[1] Lohr S: *Sampling: Design and Analysis*. Brooks/Cole 1999.

[2] Little R: **Survey Nonresponse Adjustments for Estimates of Means**. *International Statistical Review* 1986, **54**(2):139–157.

[3] Little R, Vartivarian S: **On weighting the rates in non-response weights**. *Statistics in Medicine* 2003, **22**:1589–1599.

[4] Little R: **Missing Data Adjustments in Large Surveys**. *Journal of Business and Economic Statistics* 1988, **6**(3):287–296.

[5] Mishra V, Barrere B, Hong R, Khan S: **Evaluation of bias in HIV seroprevalence estimates from national household surveys**. *Sexually Transmitted Infections* 2008, **84**(Suppl I):i63–i70.

[6] Tu X, Litvak E, Pagano M: **On the informativeness and accuracy of pooled testing in estimating prevalence of a rare disease: Application to HIV screening**. *Biometrika* 1995, **82**:287–97.

[7] Wasserman L: *All of statistics: a concise course in statistical inference*. Springer 2003.
